# Ultrasound Measurements of Rectus Femoris and Locomotor Outcomes in Patients with Spinal Cord Injury

**DOI:** 10.3390/life12071073

**Published:** 2022-07-18

**Authors:** Matthew Rong Jie Tay, Keng He Kong

**Affiliations:** 1 Department of Rehabilitation Medicine, Tan Tock Seng Hospital, Singapore 308433, Singapore; keng_he_kong@ttsh.com.sg; 2 Centre of Rehabilitation Excellence, Tan Tock Seng Hospital, Singapore 308433, Singapore; 3 Lee Kong Chian School of Medicine, Nanyang Technological University, Singapore 308232, Singapore

**Keywords:** ultrasonography, skeletal muscle, quadriceps muscle, spinal cord injuries, neurologic disorders, rehabilitation

## Abstract

Patients with incomplete spinal cord injury have decreased mobility, and many do not recover walking ability. The purpose of this study was to investigate rectus femoris muscle thickness and echo intensity on ultrasound and functional outcomes in these patients. This was a prospective cohort study in an inpatient rehabilitation center, which recruited 40 consecutive patients with incomplete spinal cord injury. The patients underwent an ultrasound assessment at 6 weeks post-injury. Ultrasound measurements were performed using B-mode ultrasound scanning and standardized protocols. Functional outcomes on discharge, including Lower Extremity Muscle Score (LEMS), Functional Independence Measure (FIM), and Walking Index for Spinal Cord Injury II (WISCI II), were measured. Rectus femoris muscle thickness was significantly correlated with discharge LEMS (Spearman’s rho = 0.448; *p* = 0.004), FIM motor subscale (Spearman’s rho = 0.595; *p* < 0.001), FIM walk subscale (Spearman’s rho = 0.621; *p* < 0.001) and WISCI II (Spearman’s rho = 0.531; *p* < 0.001). The rectus femoris echo intensity was also significantly correlated with discharge LEMS (Spearman’s rho = −0.345; *p* = 0.029), FIM motor subscale (Spearman’s rho = −0.413; *p* = 0.008), FIM walk subscale (Spearman’s rho = −0.352; *p* = 0.026), and WISCI II (Spearman’s rho = −0.355; *p* = 0.025). We report that a relationship exists between rectus femoris muscle ultrasonographic characteristics and muscle function and ambulatory outcomes after inpatient rehabilitation. Ultrasound muscle measurements are potentially useful in assessing muscle wasting and function in patients with spinal cord injury.

## 1. Introduction

Incomplete spinal cord injury leads to varying degrees of motor impairment, which often leads to decreased mobility and functional independence in affected patients [1]. Despite that, patients with spinal cord injury may recover some walking ability, depending on the capability of the lower extremity musculature to generate sufficient voluntary torque. Several imaging studies have therefore investigated the relationship between muscle structural changes and functional outcomes in these patients. For example, in patients with chronic spinal cord injury, MRI findings have demonstrated that those who were ambulators had a significantly larger plantar flexor cross-sectional area and reduced muscle fat infiltration compared to those who were wheelchair dependent, suggesting that progressive muscle wasting may result in limitations in physical function [2]. 

Although MRI has traditionally been used to assess muscle architectural changes, ultrasound has been increasingly used to assess lower extremity muscles in clinical settings due to its portability and reliability compared to MRI muscle imaging [3,4,5]. Ultrasound is also less costly and more accessible than MRI [6]. A systematic review found high reliability and validity for ultrasound in assessing muscle size in older adults, including the rectus femoris, vastus medialis, vastus intermedius, and vastus lateralis [7]. Ultrasound of the rectus femoris has also been shown to reflect muscle strength and physical performance in aging [8,9]. Similar findings have also been reported in clinical populations [10]. In healthy untrained individuals, ultrasound measurements of the quadriceps, such as cross-sectional area and echo intensity, have been reported to predict maximum muscle torque and isokinetic strength [11,12,13]. 

In patients with chronic spinal cord injury, recent ultrasonographic studies have found that lower muscle area and fatty infiltration discovered by ultrasound were associated with a lower activity level [14]. However, such studies in the literature are limited to patients with chronic spinal cord injury. To date, there are limited studies on ultrasonographic muscle architecture of patients with acute spinal cord injury. This population is of high clinical interest, as even though many of these patients are undergoing acute rehabilitation, only a small proportion achieve ambulation [15,16]. It is unclear at present if there is a relationship between ultrasonographic-derived lower extremity muscle size and fat infiltration in patients with acute spinal cord injury and the functional and ambulatory outcomes that patients achieve in the rehabilitation setting. 

The aim of this study was, therefore, to investigate the correlation between muscle thickness and intramuscular fat of the rectus femoris in acute incomplete spinal cord injury and functional outcomes. 

## 2. Methods

### 2.1. Patient Characteristics

This was a prospective single-center cohort study, recruiting a total of 40 patients with incomplete spinal cord injury admitted to the spinal cord injury unit in a tertiary rehabilitation center from 1 January 2020 to 6 June 2021. The study was conducted in accordance with the Declaration of Helsinki and was approved by the local ethics committee of the hospital (NHG DSRB 2019/00923). Informed written consent was obtained from all patients prior to their enrollment in this study.

The inclusion criteria for study participants were: first-ever acute spinal cord injury, recent onset of injury < 1 month, age of ≥21 years old, independent in ambulation prior to the onset of spinal cord injury, spinal cord injury at the cervical or thoracic levels (C4-T12) resulting in upper motor neuron lesions in the lower extremity as determined by neurological examination, and an incomplete injury determined by the American Spinal Injury Association (ASIA) Impairment Scale (AIS) C or D of sudden onset (<24 h).

The participants were excluded if they had active malignancy, premorbid lower limb musculoskeletal conditions, e.g., contractures, fractures, or previous operations, had other active neurological conditions, or were unable to understand study procedures. 

All of the spinal cord injury patients had standard inpatient rehabilitation treatment (2 h/day for 5 days/week), with daily physiotherapy and occupational therapy sessions for 1 h each session, which consisted of mobilization, gait therapy, and conventional rehabilitation. The time to mobilization was determined by the rehabilitation team based on patient assessment. The Tan Tock Seng Hospital Rehabilitation Center is a tertiary rehabilitation facility that provides comprehensive inpatient rehabilitation services for patients who are directly transferred from the acute spinal cord injury units of affiliated hospitals.

### 2.2. Ultrasound Assessment

An ultrasound assessment was performed at 6 weeks post spinal cord injury. The rectus femoris muscle was chosen due to its high level of reliability, as reported by Nijhoth et al. [7]. The rectus femoris has also been found to have low variability with respect to muscle thickness and echo intensity [17,18]. The thickness and echo intensity of the rectus femoris were measured using B-mode ultrasound scanning (Terason t3200, Terason Ultrasound, Burlington, MA, USA) with a 15–4 MHz transducer. The ultrasonographic evaluation was performed with patients in a supine position, with the hip joints in a neutral position, the knee joints in full extension, and the ankle joints in a neutral position. The probe was placed perpendicular to the skin without exerting compression, with all scans made in the transverse plane. 

The rectus femoris was measured at the mid-portion, calculated as half the distance between the anterior superior iliac spine and the lower edge of the patella [19]. 

ImageJ software (National Institute of Health, Bethesda, MD, USA, version 1.46) was used for analysis. Muscle thickness was defined as the distance between the superior border of the subcutaneous fascia and the deep aponeurosis [20]. Echo intensity was assessed via the gray scale level, which was expressed in arbitrary units (a.u.), using ImageJ software. A rectangular region of interest as large as possible was established, excluding the visible fascia and bone. The average muscle thickness and echo intensity in both lower limbs were then derived.

All of the ultrasonography measurements were performed in triplicate, with the average of the scores used in the final analyses. All of the images were obtained by the same operator.

### 2.3. Functional Scores

Muscle function was graded with the lower extremity muscle score (LEMS), which was measured on discharge in accordance with the standard neurologic assessment developed by ASIA. The voluntary muscle strength of five key muscles (hip flexors, knee extensors, ankle dorsiflexors, long toe extensors, and ankle plantar flexors) of both lower extremities was tested [21]. Each muscle was given a value between 0 and 5 according to the strength of voluntary muscle contraction. Minimum and maximum LEMS were 0 and 50, respectively.

The Functional Independence Measure (FIM) is one of the most widely used measures of disability [22]. The FIM-motor subscale was used to assess the patient’s ADL performance on admission and discharge. The FIM motor subscale ranges from 13 (totally dependent) to 91 (independent without modification). 

Gait abilities were measured using the FIM-walk subscale and the Walking Index for Spinal Cord Injury II (WISCI II). The FIM-walk subscale is scored based on the distance travelled over 150 feet and the level of assistance or device required and ranges from 1 to 7. The WISCI II assesses the physical assistance (i.e., number of people) and assistive devices (i.e., walking aids) a patient needs to ambulate 10 m, with a scale from 0 to 20, with a higher number indicating less impairment [23]. 

### 2.4. Statistical Analysis

The distributions of the sociodemographic and clinical data were presented with appropriate descriptive statistics, e.g., standard deviation. The Student’s *t*-test or analysis of variance (ANOVA) was used for the analysis of the continuous variables for the independent samples as appropriate when examining the effect of age and ethnicity on ultrasound measurements. The correlation between the muscle thickness and echo intensity of the rectus femoris with clinical characteristics (age, height, and weight) as well as the outcome variables (FIM motor subscale, FIM walk subscale, LEMS, WISCI II) were determined using Spearman rank correlation coefficient. Poor, fair, moderately strong, and very strong correlation coefficients were defined as <0.3, 0.3–0.5, 0.60–0.80, and at least 0.80, respectively [24]. Statistical analyses were performed using SPSS version 26.0 (IBM Corp., Armonk, NY, USA). All statistical tests were performed at a two-sided 5% significance level. 

## 3. Results

There were 40 patients recruited, with two patients excluded due to the presence of lower limb fractures. The mean age of 60.0 ± 16.7 years. The majority of spinal cord injury etiologies were due to falls (65.0%) and motor vehicle accidents (25.0%). The study participants had either ASIA C (40.0%) or ASIA D (60.0%) injuries. The characteristics of the study cohort are shown in Table 1. 

On discharge from inpatient rehabilitation, the participants had a mean LEMS of 35.2 ± 17.3, FIM motor subscale of 50.2 ± 25.7, FIM walk subscale of 3.90 ± 2.19, and a WISCI II of 10.2 ± 7.56. On the ultrasound assessment performed, the study participants were found to have a mean rectus femoris thickness of 137.3 ± 54.3 mm and a mean rectus femoris echo intensity of 65.7 ± 24.7 AU at 6 weeks post-injury. 

There were no significant correlations between the clinical characteristics (age, height, weight, gender, ethnicity) and the ultrasound measurements of the rectus femoris muscle. 

After performing correlation analyses, the rectus femoris muscle thickness was found to be positively correlated with LEMS (Spearman’s rho = 0.448; *p* = 0.004), FIM motor subscale (Spearman’s rho = 0.595; *p* < 0.001), FIM walk subscale (Spearman’s rho = 0.621; *p* < 0.001), and WISCI II (Spearman’s rho = 0.531; *p* < 0.001) (Table 2).

We also found that the rectus femoris echo intensity was negatively correlated with LEMS (Spearman’s rho = −0.345; *p* = 0.029), FIM motor subscale (Spearman’s rho = −0.413; *p* = 0.008), FIM walk subscale (Spearman’s rho = −0.352; *p* = 0.026), and WISCI II (Spearman’s rho = −0.355; *p* = 0.025) (Table 2).

## 4. Discussion

We report fair to moderately strong correlations between ultrasound muscle architecture of the rectus femoris muscle and functional outcomes in patients with incomplete spinal cord injury. 

Studies in non-spinal cord injury populations have demonstrated that ultrasonographic muscle changes may have a significant relationship with functional measures. An observational study in patients with subacute stroke undergoing rehabilitation reported findings of decreased muscle mass and increased echo intensity in the vastus lateralis [25]. Reduced quadriceps muscle thickness in patients with subarachnoid hemorrhage was found to be correlated with a poorer modified Rankin Scale at 90 days post-stroke [26]. In patients with chronic stroke, echo intensity and muscle thickness of the quadriceps were found to be related to muscle strength and a decrease in aerobic exercise capacity [27,28]. Apart from patients with stroke, studies in older adults have found correlations between lower limb muscle thickness, knee extensor strength and physical activity [29,30]. A high echo intensity on muscle ultrasound has also been shown to be correlated with lower muscle quality and grip strength in older adults [31,32]. Other studies in older patients have also reported an association of echo intensity with muscle power [33], gait ability [34], and Short Physical Performance Battery [35]. This study mirrors the findings in non-spinal cord injury patients, suggesting that both muscle size and muscle quality evaluated by ultrasonography can be a useful marker of functional capacity. 

Few studies have examined lower extremity muscle thickness in patients with acute incomplete spinal cord injury. Although a case series of six patients with incomplete spinal cord injury showed a reduction in muscle cross-sectional area at 6 weeks compared to healthy controls [36], no functional correlates were reported. We report a significant correlation between the lower extremity muscle thickness in patients with incomplete spinal cord injury and functional scores in terms of LEMS, FIM motor, FIM walk, and WISCI II scores. Muscle thickness is believed to be a reproducible measure of muscle mass and hence has been thought to influence functional measures [37,38,39]. One possible explanation for our findings is that the participants who had a higher LEMS or ambulatory score were able to weight-bear through the affected lower extremities, activating the anti-gravity lower extremity muscles and attenuating the degree of muscle atrophy, and hence had a larger muscle thickness. This is supported by studies that demonstrated that an increased cross-sectional area of quadriceps on MRI and improved lean body mass on DEXA after therapeutic exercise with functional electrical stimulation or body weight supported treadmill training in spinal cord injury patients [40]. Our findings, therefore, suggest that ultrasound-derived measurements of muscle size, as a surrogate of muscle strength, is potentially a marker of ambulatory performance. 

We also found reduced echo intensity, which indicates reduced intramuscular fat, to be correlated with superior muscle function and ambulatory outcomes. Although accumulation of intramuscular fat after chronic spinal cord injury has been well studied [41], there are no studies that have investigated functional correlates in acute spinal cord injury. For example, Moore et al. found that LEMS was associated with increased muscle density as measured on CT for both complete and incomplete chronic spinal cord injury patients, although no ambulatory outcomes were obtained [14]. Studies in the non-neurologically impaired population have also reported a negative relationship between fatty infiltration, as evaluated by ultrasonographic echo intensity, with functional performance in middle-aged and older individuals [42]. It has also been postulated that prolonged critical illness and sedentary behavior can result in ectopic fat accumulation in skeletal muscles [43,44,45]. We believe that our results build on these prior studies by demonstrating that increased echo intensity is correlated with muscle function and ambulatory outcomes in the acute phase. Fukumoto et al. showed that skeletal muscle echo intensity is associated with intramuscular infiltration of adipose tissue [29], and ultrasound measurements provide a reliable method for evaluating muscle health. Hence, measuring muscle echo intensity may be a useful adjunct for measuring skeletal muscle function and guiding rehabilitation interventions in the subacute phase. 

We did not find any significant correlation with clinical characteristics in the acute spinal cord injury setting. A negative relationship between age and a loss of muscle mass and increased intramuscular fat has been reported in healthy adults [46]. Weight has also been thought to contribute to muscle thickness and echo intensity in chronic stroke survivors [47]. However, these were not found in our study in patients with acute spinal cord injury, possibly because our patients were relatively older and had a higher body mass index. We speculate that acute muscle wasting may also have played a more significant role compared to the effects of age or body mass index. 

Some limitations in this study should be highlighted. Firstly, we did not measure other muscle function outcomes such as muscle strength or torque. Second, although we studied muscle parameters on discharge, we did not study if longitudinal changes in the rectus femoris parameters were also a determinant of functional outcomes. We also did not measure the extent of spasticity, which may affect muscle echo intensity [48]. Third, although ultrasound echo intensity measurements are reliable, the lack of comparison with absolute fat mass as measured on DEXA or intramuscular fat infiltration as measured on MRI was also a limitation. Fourth, we did not study the other muscles of the quadriceps, nor did we compare measurements at proximal or distal regions of the thigh [17]. Fifth, we also did not correct for subcutaneous fat when measuring muscle echo intensity [18]. Lastly, asymmetric lower limb neurological deficits may have differential effects on the muscle thickness and echo intensity of either limb, although we did not study the contribution of the left or right rectus femoris in isolation.

## 5. Conclusions

In conclusion, our findings suggest that rectus femoris muscle thickness and echo intensity may be a valid method to assess muscle function and ambulatory outcomes during inpatient rehabilitation. These findings have the potential to guide rehabilitative interventions targeted at the muscle unit to induce muscle hypertrophy and architectural changes [49]. Further prospective studies are required to determine the contributory factors to muscular atrophy and fatty infiltration during the process of spinal cord rehabilitation.

## Figures and Tables

**Table 1:** Participants’ characteristics (*n* = 40).

Characteristics	
Age, years, mean ± SD	60.0 ± 16.7
Sex (Male: female)	29:11
Ethnicity, *n* (%)	
Chinese	31 (77.5)
Malay	8 (20.0)
Indian	1 (2.5)
Etiology, *n* (%)	
Fall	26 (65.0)
Motor vehicle accident	10 (25.0)
Infectious	1 (2.5)
Inflammatory	2 (5.0)
Vascular	1 (2.5)
ASIA classification, *n* (%)	
C	16 (40.0)
D	24 (60.0)
Height, mean ± SD	1.65 ± 0.0904
Weight, mean ± SD	62.4 ± 17.8
Body mass index, mean ± SD	22.9 ± 5.52
Average rehabilitation stay, mean ± SD	57.3 ± 10.2
Admission scores	
LEMS, mean ± SD	28.4 ± 17.0
FIM motor subscale, mean ± SD	29.4 ± 15.0
FIM walk subscale, mean ± SD	1.78 ± 1.12
WISCI II, mean ± SD	4.25 ± 6.00
Discharge scores	
LEMS, mean ± SD	35.2 ± 17.3
FIM motor subscale, mean ± SD	50.2 ± 25.7
FIM walk subscale, mean ± SD	3.90 ± 2.19
WISCI II, mean ± SD	10.2 ± 7.56
Rectus femoris thickness, mm	137.3 (54.3)
Rectus femoris echo intensity, AU	65.7 (24.7)

ASIA: American Spinal Cord Injury Association; LEMS: Lower Extremity Motor Score; FIM: Functional Independence Measure; WISCI II: Walking Index for Spinal Cord Injury II, AU: Arbitrary Units.

**Table 2:** Correlation coefficients between rectus femoris muscle thickness and echo intensity with discharge functional outcomes.

Outcome Variables	Correlation Coefficient (Spearman’s Rho)	*p* Value
Rectus femoris muscle thickness		
LEMS	0.448	0.004
FIM motor subscale	0.595	<0.001
FIM walk subscale	0.621	<0.001
WISCI II	0.531	<0.001
Rectus femoris echo intensity		
LEMS	−0.345	0.029
FIM motor subscale	−0.413	0.008
FIM walk subscale	−0.352	0.026
WISCI II	−0.355	0.025

LEMS: Lower Extremity Motor Score; FIM: Functional Independence Measure; WISCI II: Walking Index for Spinal Cord Injury II.

## Data Availability

The data presented in this study are available on request from the corresponding author.

## References

[1] Sinovas-Alonso I., Gil-Agudo Á., Cano-de-la-Cuerda R., Del-Ama A.J. (2021). Walking Ability Outcome Measures in Individuals with Spinal Cord Injury: A Systematic Review. Int. J. Environ. Res. Public Health.

[2] Shah P.K., Stevens J.E., Gregory C.M., Pathare N.C., Jayaraman A., Bickel S.C., Bowden M., Behrman A.L., Walter G.A., Dudley G.A. (2006). Lower-extremity muscle cross-sectional area after incomplete spinal cord injury. Arch. Phys. Med. Rehabil..

[3] Smith A.C., Jakubowski K., Wasielewski M., Lee S.S., Elliott J.M. (2017). Lower extremity muscle structure in incomplete spinal cord injury: A comparison between ultrasonography and magnetic resonance imaging. Spinal Cord Ser. Cases.

[4] Berger J., Bunout D., Barrera G., de la Maza M.P., Henriquez S., Leiva L., Hirsch S. (2015). Rectus femoris (RF) ultrasound for the assessment of muscle mass in older people. Arch. Gerontol. Geriatr..

[5] Raj I.S., Bird S.R., Shield A.J. (2012). Reliability of ultrasonographic measurement of the architecture of the vastus lateralis and gastrocnemius medialis muscles in older adults. Clin. Physiol. Funct. Imaging.

[6] Reeves N.D., Maganaris C.N., Narici M.V. (2004). Ultrasonographic assessment of human skeletal muscle size. Eur. J. Appl. Physiol..

[7] Nijholt W., Scafoglieri A., Jager-Wittenaar H., Hobbelen J.S.M., van der Schans C.P. (2017). The reliability and validity of ultrasound to quantify muscles in older adults: A systematic review. J. Cachexia Sarcopenia Muscle.

[8] Nies I., Ackermans L.L.G.C., Poeze M., Blokhuis T.J., Bosch J.T. (2022). The Diagnostic Value of Ultrasound of the Rectus Femoris for the diagnosis of Sarcopenia in adults: A systematic review. Injury.

[9] Ata A.M., Kara M., Kaymak B., Gürçay E., Çakır B., Ünlü H., Akıncı A., Özçakar L. (2019). Regional and total muscle mass, muscle strength and physical performance: The potential use of ultrasound imaging for sarcopenia. Arch. Gerontol. Geriatr..

[10] English C., Fisher L., Thoirs K. (2012). Reliability of real-time ultrasound for measuring skeletal muscle size in human limbs in vivo: A systematic review. Clin. Rehabil..

[11] Trezise J., Blazevich A.J. (2019). Anatomical and Neuromuscular Determinants of Strength Change in Previously Untrained Men Following Heavy Strength Training. Front. Physiol..

[12] Oranchuk D.J., Hopkins W.G., Nelson A.R., Storey A.G., Cronin J.B. (2021). The effect of regional quadriceps anatomical parameters on angle-specific isometric torque expression. Appl. Physiol. Nutr. Metab..

[13] Yamauchi T., Yamada T., Satoh Y. (2021). Relationship between muscle echo intensity on ultrasound and isokinetic strength of the three superficial quadriceps femoris muscles in healthy young adults. J. Phys. Ther. Sci..

[14] Moore C.D., Craven B.C., Thabane L., Laing A.C., Frank-Wilson A.W., Kontulainen S.A., Papaioannou A., Adachi J.D., Giangregorio L.M. (2015). Lower-extremity muscle atrophy and fat infiltration after chronic spinal cord injury. J. Musculoskelet. Neuronal Interact..

[15] Kirshblum S.C., Priebe M.M., Ho C.H., Scelza W.M., Chiodo A.E., Wuermser L.A. (2007). Spinal cord injury medicine: 3—Rehabilitation phase after acute spinal cord injury. Arch. Phys. Med. Rehabil..

[16] van Middendorp J.J., Hosman A.J., Donders A.R., Pouw M.H., Ditunno J.F., Curt A., Geurts A.C., Van de Meent H., EM-SCI Study Group (2011). A clinical prediction rule for ambulation outcomes after traumatic spinal cord injury: A longitudinal cohort study. Lancet.

[17] Oranchuk D.J., Nelson A.R., Storey A.G., Cronin J.B. (2019). Variability of Regional Quadriceps Architecture in Trained Men Assessed by B-Mode and Extended-Field-of-View Ultrasonography. Int. J. Sports Physiol. Perform..

[18] Oranchuk D.J., Stock M.S., Nelson A.R., Storey A.G., Cronin J.B. (2020). Variability of regional quadriceps echo intensity in active young men with and without subcutaneous fat correction. Appl. Physiol. Nutr. Metab..

[19] Arts I.M., Pillen S., Schelhaas H.J., Overeem S., Zwarts M.J. (2010). Normal values for quantitative muscle ultrasonography in adults. Muscle Nerve.

[20] De Boer M.D., Seynnes O.R., di Prampero P.E., Pisot R., Mekjavić I.B., Biolo G., Narici M.V. (2008). Effect of 5 weeks horizontal bed rest on human muscle thickness and architecture of weight bearing and non-weight bearing muscles. Eur. J. Appl. Physiol..

[21] Kirshblum S.C., Burns S.P., Biering-Sorensen F., Donovan W., Graves D.E., Jha A., Johansen M., Jones L., Krassioukov A., Mulcahey M.J. (2011). International standards for neurological classification of spinal cord injury (revised 2011). J. Spinal Cord Med..

[22] Hamilton B.B., Laughlin J.A., Fiedler R.C., Granger C.V. (1994). Interrater reliability of the 7-level functional independence measure (FIM). Scand. J. Rehabil. Med..

[23] Dittuno P.L., Ditunno J.F. (2001). Walking index for spinal cord injury (WISCI II): Scale revision. Spinal Cord.

[24] Chan Y.H. (2003). Biostatistics 104: Correlational analysis. Singapore Med. J..

[25] Kim J.M., Tay M.R.J., Rajeswaran D.K., Tham S.L., Lui W.L., Kong K.H. (2021). Changes in muscle architecture on ultrasound in patients early after stroke. NeuroRehabilitation.

[26] Nozoe M., Kanai M., Kubo H., Kobayashi M., Yamamoto M., Shimada S., Mase K. (2018). Quadriceps muscle thickness changes in patients with aneurysmal subarachnoid hemorrhage during the acute phase. Top. Stroke Rehabil..

[27] Akazawa N., Harada K., Okawa N., Tamura K., Moriyama H. (2018). Muscle mass and intramuscular fat of the quadriceps are related to muscle strength in non-ambulatory chronic stroke survivors: A cross-sectional study. PLoS ONE.

[28] Ryan A.S., Dobrovolny C.L., Silver K.H., Smith G.V., Macko R.F. (2000). Cardiovascular fitness after stroke: Role of muscle mass and gait deficit severity. J. Stroke Cerebrovasc. Dis..

[29] Fukumoto Y., Ikezoe T., Yamada Y., Tsukagoshi R., Nakamura M., Mori N., Kimura M., Ichihashi N. (2012). Skeletal muscle quality assessed from echo intensity is associated with muscle strength of middle-aged and elderly persons. Eur. J. Appl. Physiol..

[30] Herrick I., Brown S., Agyapong-Badu S., Warner M., Ewings S., Samuel D., Stokes M. (2017). Anterior Thigh Tissue Thickness Measured Using Ultrasound Imaging in Older Recreational Female Golfers and Sedentary Controls. Geriatrics.

[31] Reimers K., Reimers C., Wagner S., Paetzke I., Rer Nat D., Pongratz D. (1993). Skeletal muscle sonography: A correlative study of echogenicity and morphology. J. Ultrasound Med..

[32] Ismail C., Zabal J., Hernandez H.J., Woletz P., Manning H., Teixeira C., DiPietro L., Blackman M.R., Harris-Love M.O. (2015). Diagnostic ultrasound estimates of muscle mass and muscle quality discriminate between women with and without sarcopenia. Front. Physiol..

[33] Hobson-Webb L.D., Zwelling P.J., Pifer A.N., Killelea C.M., Faherty M.S., Sell T.C., Pastva A.M. (2018). Point of Care Quantitative Assessment of Muscle Health in Older Individuals: An Investigation of Quantitative Muscle Ultrasound and Electrical Impedance Myography Techniques. Geriatrics.

[34] Rech A., Radaelli R., Goltz F.R., da Rosa L.H.T., Schneider C.D., Pinto R.S. (2014). Echo Intensity is Negatively Associated with Functional Capacity in Older Women. AGE.

[35] Mateos-Angulo A., Galán-Mercant A., Cuesta-Vargas A.I. (2021). Muscle Thickness and Echo Intensity by Ultrasonography and Cognitive and Physical Dimensions in Older Adults. Diagnostics.

[36] Gorgey A.S., Dudley G.A. (2007). Skeletal muscle atrophy and increased intramuscular fat after incomplete spinal cord injury. Spinal Cord.

[37] Wang J.C., Wu W.T., Chang K.V., Chen L.R., Chi S.Y., Kara M., Özçakar L. (2021). Ultrasound Imaging for the Diagnosis and Evaluation of Sarcopenia: An Umbrella Review. Life.

[38] Dos Santos L.P., do Espírito Santo R.C., Pena É., Dória L.D., Hax V., Brenol C.V., Monticielo O.A., Chakr R.M.D.S., Xavier R.M. (2021). Morphological Parameters in Quadriceps Muscle Were Associated with Clinical Features and Muscle Strength of Women with Rheumatoid Arthritis: A Cross-Sectional Study. Diagnostics.

[39] Leigheb M., de Sire A., Colangelo M., Zagaria D., Grassi F.A., Rena O., Conte P., Neri P., Carriero A., Sacchetti G.M. (2021). Sarcopenia Diagnosis: Reliability of the Ultrasound Assessment of the Tibialis Anterior Muscle as an Alternative Evaluation Tool. Diagnostics.

[40] Panisset M.G., Galea M.P., El-Ansary D. (2015). Does early exercise attenuate muscle atrophy or bone loss after spinal cord injury?. Spinal Cord.

[41] Biering-Sørensen B., Kristensen I.B., Kjaer M., Biering-Sørensen F. (2009). Muscle after spinal cord injury. Muscle Nerve.

[42] Roubenoff R., Hughes V.A. (2000). Sarcopenia: Current concepts. J. Gerontol. Ser. A Biol. Sci. Med. Sci..

[43] Chung G.E., Park H.E., Kim M.J., Kwak M.S., Yang J.I., Chung S.J., Yim J.Y., Yoon J.W. (2021). The Association between Low Muscle Mass and Hepatic Steatosis in Asymptomatic Population in Korea. Life.

[44] Cho A.R., Lee J.H., Kwon Y.J. (2021). Differences among Three Skeletal Muscle Mass Indices in Predicting Non-Alcoholic Fatty Liver Disease: Korean Nationwide Population-Based Study. Life.

[45] Klawitter F., Walter U., Patejdl R., Endler J., Reuter D.A., Ehler J. (2022). Sonographic Evaluation of Muscle Echogenicity for the Detection of Intensive Care Unit-Acquired Weakness: A Pilot Single-Center Prospective Cohort Study. Diagnostics.

[46] Fukumoto Y., Ikezoe T., Yamada Y., Tsukagoshi R., Nakamura M., Takagi Y., Kimura M., Ichihashi N. (2015). Age-related ultrasound changes in muscle quantity and quality in women. Ultrasound Med. Biol..

[47] Akazawa N., Harada K., Okawa N., Tamura K., Moriyama H. (2019). Low body mass index negatively affects muscle mass and intramuscular fat of chronic stroke survivors. PLoS ONE.

[48] Cosenza L., Picelli A., Azzolina D., Minetto M.A., Invernizzi M., Bertoni M., Santamato A., Baricich A. (2020). Rectus Femoris Characteristics in Post Stroke Spasticity: Clinical Implications from Ultrasonographic Evaluation. Toxins.

[49] Jandova T., Narici M.V., Steffl M., Bondi D., D’Amico M., Pavlu D., Verratti V., Fulle S., Pietrangelo T. (2020). Muscle Hypertrophy and Architectural Changes in Response to Eight-Week Neuromuscular Electrical Stimulation Training in Healthy Older People. Life.

